# Interventions aimed at increasing research use in nursing: a systematic review

**DOI:** 10.1186/1748-5908-2-15

**Published:** 2007-05-11

**Authors:** David S Thompson, Carole A Estabrooks, Shannon Scott-Findlay, Katherine Moore, Lars Wallin

**Affiliations:** 1 Knowledge Utilization Studies Program, Faculty of Nursing, 5-112 Clinical Sciences Building, University of Alberta, Edmonton, Alberta, T6G 2G3 Canada; 2 Faculty of Nursing, 5-112 Clinical Sciences Building, University of Alberta, Edmonton, Alberta, T6G 2G3 Canada; 3 Department of Pediatrics and Centre for Health Promotion Studies, Room 9432, 4th Floor, Aberhart Centre One, 11402 University Avenue, University of Alberta, Edmonton, Alberta, T6G 2J3 Canada; 4 Faculty of Nursing, 3rd Floor, Clinical Sciences Building University of Alberta, Edmonton, Alberta, T6G 2G3 Canada; 5 Karolinska University Hospital, Eugeniahemmet T4:02 SE-171 76, Stockholm, Sweden

## Abstract

**Background:**

There has been considerable interest recently in developing and evaluating interventions to increase research use by clinicians. However, most work has focused on medical practices; and nursing is not well represented in existing systematic reviews. The purpose of this article is to report findings from a systematic review of interventions aimed at increasing research use in nursing.

**Objective:**

To assess the evidence on interventions aimed at increasing research use in nursing.

**Methods:**

A systematic review of research use in nursing was conducted using databases (Medline, CINAHL, Healthstar, ERIC, Cochrane Central Register of Controlled Trials, and Psychinfo), grey literature, ancestry searching (Cochrane Database of Systematic Reviews), key informants, and manual searching of journals. Randomized controlled trials and controlled before- and after-studies were included if they included nurses, if the intervention was explicitly aimed at increasing research use or evidence-based practice, and if there was an explicit outcome to research use. Methodological quality was assessed using pre-existing tools. Data on interventions and outcomes were extracted and categorized using a pre-established taxonomy.

**Results:**

Over 8,000 titles were screened. Three randomized controlled trials and one controlled before- and after-study met the inclusion criteria. The methodological quality of included studies was generally low. Three investigators evaluated single interventions. The most common intervention was education. Investigators measured research use using a combination of surveys (three studies) and compliance with guidelines (one study). Researcher-led educational meetings were ineffective in two studies. Educational meetings led by a local opinion leader (one study) and the formation of multidisciplinary committees (one study) were both effective at increasing research use.

**Conclusion:**

Little is known about how to increase research use in nursing, and the evidence to support or refute specific interventions is inconclusive. To advance the field, we recommend that investigators: (1) use theoretically informed interventions to increase research use, (2) measure research use longitudinally using theoretically informed and psychometrically sound measures of research use, as well as, measuring patient outcomes relevant to the intervention, and (3) use more robust and methodologically sound study designs to evaluate interventions. If investigators aim to establish a link between using research and improved patient outcomes they must first identify those interventions that are effective at increasing research use.

## Background

Nurses constitute the largest group of health care providers and their care influences patient outcomes [1-3]. However, nurses, like other professionals, often fail to incorporate current research findings into their practices [4]. A lack of research use contributes to as many as 30%–40% of patients not receiving care, according to current scientific evidence, and some 20%–25% of patients may receive potentially harmful care [5]. In response, much attention has been directed to developing interventions aimed at changing provider behavior to reflect current research. Several systematic reviews have been published in this area [6-10], and authors of such reviews primarily include physicians and outcomes relevant to physicians. For example, Grimshaw and colleagues included only medical providers in a systematic review of guideline dissemination strategies [8]. Additionally, in a review of continuing education meetings and workshops, only four of the thirty-two studies included nurses [9]. Poor representation of nursing studies in existing reviews is partially a result of a lack of rigorous nursing research in the area of research utilization. For example, in a review of organizational infrastructures aimed at increasing evidence-based nursing practice, Foxcroft and Cole could locate no studies rigorous enough to be included [11].

Generalizing findings from existing reviews to nursing is problematic. While physicians and nurses experience similar challenges in incorporating evidence, there are differences that influence how each group uses research in practice. One key issue is the social structure of the two professions. Nurses typically work in hierarchical social structures as salaried employees. Conversely, in many countries physicians typically work in more autonomous group practices or in hospitals, not as salaried employees, but as attending physicians with privileges [12]. In these configurations, with the different resulting relationships with the organization, it is likely organizational context will exert different influences on the two groups. A second key difference, related to inpatient care, is the nature and structure of the work of the two professions. Nursing is typically responsible for continuous care over a short period of time. Conversely, episodic contact, often of longer duration, is more the case with medical practice. Moreover, nursing practice does not typically include medical diagnosis or prescribing of diagnostic or therapeutic interventions (although this is changing with the movement to nurse practitioners and other extended practice nursing roles). While these differences are not as common beyond inpatient settings (i.e., community care), the majority of nursing care continues to be provided in hospital settings. Therefore, results from existing reviews cannot be assumed to transfer readily or well to nursing practice in general.

Another weakness, we argue, with existing literature is investigators' reliance upon provider behavior change as a proxy for research use. For example, 88.8% of studies included in a widely cited and influential systematic review of studies aimed at increasing evidence-based practice used behavior practice changes as outcome measures [13]. Using provider behavior as a proxy for research use has some limitations.

First, relating to different meanings of *research use*, scholars generally accept three forms of research utilization: instrumental, conceptual and symbolic [14-17]. Instrumental research utilization is the concrete application of research in practice [15,17]. Most often, this involves using research to carry out an actionable behavior. Conceptual research utilization is the use of research to change one's thinking but not necessarily one's action [15,17]. Symbolic research utilization refers to the use of research to influence policies or decisions [15,17]. Investigators have shown the three forms of research utilization can be measured with self-report questionnaires [14,17-20]. However, authors of existing studies (and reviews) have relied primarily upon behavior change outcomes [13]. Because instrumental research use results in actionable behavior while conceptual and symbolic may not, measuring behavior change may only capture instrumental research use – a portion of the larger research utilization construct.

Second, research in our group has focused on more general measures of research utilization as opposed to specific guidelines or innovation-specific measures. Specific guideline measures have an important role in the understanding of the influences on research uptake, and they permit identification of guideline characteristics that may differentially influence reports of research use. However, we lack direction when attempting to ascertain a level of uptake that can be considered representative of a *patient care unit *or *organization*, or when seeking a formula with which to derive a unit or organization's level of research uptake. Thus researchers at organizational levels must rely on the very general measures identified above. Our experience with these general measures has been reasonably promising – we are able to capture variance in responses, the responses are reasonably normally distributed, and factors that one would expect to predict research utilization have generally held true.

Third, while research utilization is assumed to have a positive impact on patient outcomes through provider behavior, this is poorly understood and the means by which this occurs is believed to be inconsistent and complex [21]. The process by which research becomes used in practice has, in fact, been treated as something of a 'black box phenomenon' [22]. We know that providers base their behavior on many mediating factors, one of which may be research findings [21,23]. Factors such as professional training, clinician experience, organizational context, and administrative support are also influential. Drawing conclusions about the effectiveness of research utilization interventions based on changes in provider behavior alone is probably an unreliable approach, because it is not clear how much of a behavior change can be ascribed to research use and how much to other factors. If provider behavior change results in a patient, or other outcome change, investigators are unable to determine if this is a direct effect (of provider behavior on patient outcome) or an indirect effect, that is, an effect mediated by research utilization. If it is the latter, then understanding which factors are mediated *via *a research utilization variable is important as the causal forces that are exerted on that variable may themselves be modifiable but would remain undetected if only behavior change were measured.

The aim of this systematic review was to assess the evidence on interventions aimed explicitly at increasing research use in nursing practice. We were interested in reports in which the investigators had explicitly measured research use. We were therefore interested explicitly in studies that used some general measure of research use.

## Methods

### Search Strategy

In consultation with a Library Information Specialist familiar with the field, we searched Medline, CINAHL, Healthstar, ERIC, Cochrane Central Register of Controlled Trials, and Psychinfo from inception to February 2006 (Table 1). Ancestry searches were conducted on relevant studies, and systematic reviews indexed in the Cochrane Database of Systematic Reviews and elsewhere [6-11]. We searched grey literature using the System for Information on Grey Literature database (SIGLE), the New York Academy of Medicine, and the Sarah Cole Hirsch Institute. We retrieved the majority of relevant studies from our database search from the *Journal of Nursing Care Quality*, *MEDSURG Nursing*, *Journal of Clinical Nursing *and *Journal of Gerontological Nursing*. We manually searched these journals from 1990 (or their inception) to 2006.

**Table 1:** Search strategy

CINAHL (1982-February 2006)
1. exp NURSING CARE/
2. exp NURSES/
3. exp Practice Guidelines/
4. exp AUDIOVISUALS/
5. exp PAMPHLETS/
6. exp "POLICY AND PROCEDURE MANUALS"/
7. exp Nursing Protocols/
8. exp Staff Development/
9. inservice$.mp.
10. exp "Seminars and Workshops"/
11. exp Education, Clinical/
12. exp Clinical Nurse Specialists/
13. exp Nurse Practitioners/
14. exp Staff Development Instructors/
15. exp Nurse Consultants/
16. (chang$ adj2 agent$).mp.
17. (facilitat$ adj2 change$).mp.
18. (coordinat$ adj2 change$).mp.
19. exp Quality Assurance/
20. (critical adj1 appraisal).mp.
21. exp Quality Improvement/
22. exp Reminder Systems/
23. (champion$ adj1 change$).mp.
24. exp "Diffusion of Innovation"/
25. exp Nursing Practice, Research-Based/
26. evidence based nursing.mp.
27. (utilizat$ or utilisa$ or uptake or transfer$ or implement$ or disseminat$ or diffusion$ or translat$).mp.
28. journal club.mp.
29. exp Nursing Practice, Evidence-Based/
30. 1 or 2
31. or/3–23
32. 31 or 28
33. or/24–27
34. 33 or 29
35. 30 and 32 and 34
36. limit 35 to research

Medline (1966-February 2006)
1. exp NURSING/
2. exp NURSES/
3. exp Practice Guidelines/
4. exp AUDIOVISUAL AIDS/
5. exp PAMPHLETS/
6. exp MANUALS/
7. exp CLINICAL PROTOCOLS/
8. exp Inservice Training/
9. seminar.mp.
10. workshop.mp.
11. clinical education.mp.
12. exp Nurse Clinicians/
13. clinical nurse specialist$.mp.
14. exp Nurse Practitioners/
15. nurse educator$.mp.
16. staff instructor$.mp.
17. exp Consultants/
18. exp Nurse Clinicians/
19. (chang$ adj2 agent$).mp.
20. (facilitator$ adj2 chang$).mp.
21. (coordinator$ adj2 chang$).mp.
22. (champion$ adj2 chang$).mp.
23. journal club.mp.
24. exp Quality Assurance, Health Care/
25. exp REMINDER SYSTEMS/
26. exp "Diffusion of Innovation"/
27. exp Evidence-Based Medicine/
28. exp Nursing Research/
29. (utilizat$ or utlisat$ or uptake or transfer$ or implement$ or disseminat$ or diffusion$ or translat$).mp.
30. 1 or 2
31. or/3–25
32. or/26–29
33. 30 and 31 and 32

PsychINFO (1887-February 2006)
exp NURSING/
2. exp NURSES/
3. exp Treatment Guidelines/
4. exp EDUCATIONAL AUDIOVISUAL AIDS/
5. pamphlets.mp.
6. (policy and procedure).mp. [mp = title, abstract, subject headings, table of contents, key concepts]
7. protocol.mp.
8. exp Professional Development/
9. inservice.mp.
10. workshop.mp.
11. seminar.mp.
12. clinical nurse specialist.mp.
13. nurse practitioner.mp.
14. instructor.mp.
15. nurse consultant.mp.
16. (chang$ adj2 agent$).mp.
17. (facilitat$ adj2 chang$).mp.
18. (coordinat$ adj2 change).mp.
19. exp "Quality of Services"/
20. (critical adj1 appraisal).mp.
21. reminder$.mp.
22. (champion$ adj1 change$).mp.
23. diffusion of innovation.mp.
24. exp Decision Making/
25. (research and (utiliz$ or utilis$ or uptake or transfer or implement$ or disseminat$ or translat$)).mp. [mp = title, abstract, subject headings, table of contents, key concepts]
26. (knowledge and (utiliz$ or utilis$ or uptake or transfer or implement$ or disseminat$ or translat$)).mp. [mp = title, abstract, subject headings, table of contents, key concepts]
27. (evidence adj1 practice).mp.
28. journal club.mp.
29. 1 or 2
30. or/2–22
31. 30 or 28
32. or/23–27
33. 29 and 31 and 32

HealthSTAR/Non-medlie (1975-February 2006)
1.exp NURSING/
2. exp NURSES/
3. exp Practice Guidelines/
4. exp AUDIOVISUAL AIDS/
5. exp PAMPHLETS/
6. exp MANUALS/
7. exp CLINICAL PROTOCOLS/
8. exp Inservice Training/
9. seminar.mp.
10. workshop.mp.
11. clinical education.mp.
12. exp Nurse Clinicians/
13. clinical nurse specialist$.mp.
14. exp Nurse Practitioners/
15. nurse educator$.mp.
16. staff instructor$.mp.
17. exp Consultants/
18. exp Nurse Clinicians/
19. (chang$ adj2 agent$).mp.
20. (facilitator$ adj2 chang$).mp.
21. (coordinator$ adj2 chang$).mp.
22. (champion$ adj2 chang$).mp.
23. journal club.mp.
24. exp Quality Assurance, Health Care/
25. exp REMINDER SYSTEMS/
26. exp "Diffusion of Innovation"/
27. exp Evidence-Based Medicine/
28. exp Nursing Research/
29. (utilizat$ or utlisat$ or uptake or transfer$ or implement$ or disseminat$ or diffusion$ or translat$).mp.
30. 1 or 2
31. or/3–25
32. or/26–29
33. 30 and 31 and 32
34. limit 33 to nonmedline

ERIC (1966-February 2006)
1. nurs*.tx
2. (practice guidelines).tx
3. audiovisual.tx
4. (policy and procedure).tx
5. protocol*.tx
6. (staff development).tx
7. (in service).tx
8. seminar.tx
9. workshop.tx
10.(journal club).tx
11.(clinical education).tx
12. (clinical nurse specialist).tx
13.(nurse practitioner).tx
14.instructor.tx
15.consultant.tx
16.(change agent).tx
17.champion.tx
18.coordinator.tx
19.facilitator.tx
20.(clinical educator).tx
21.(quality assurance).tx
22.(critical appraisal).tx
23.(quality improvement).tx
24.(reminder).tx
25.or/2–24
26. 1 and 25

### Inclusion Criteria

A study was eligible for inclusion if: 1) it was a randomized controlled trial (RCT) or controlled before and after (CBA) design, 2) authors evaluated interventions aimed at increasing research use or evidence-based practice, 3) participants were nurses, and 4) outcomes directly and explicitly captured research use. Only studies in English were assessed.

For criterion one, we defined RCT and CBA using Cochrane definitions. To meet criterion two, investigators must have explicitly stated that the research purpose was to test an intervention aimed at increasing research or evidence-based practice. For criterion three, we included both registered and student nurses and did not exclude based on type of nurse (i.e., psychiatric nurse, license practical nurse, etc). However, we did not include studies of nurse practitioners because, we argue, their practice has more similarities to medical practice than nursing. To meet criterion four, investigators must have explicitly described how their chosen outcomes represented research use or have used an instrument designed explicitly to measure research use. We excluded studies unless authors were explicitly clear as to how chosen outcomes captured a conceptualization of research use. This was a clear decision when authors used a tool designed to measure research use. However, to be included when a change in provider behavior was the outcome, the investigator had to have clearly described how the behavior reflected research use. For example, in evaluating the implementation of a clinical practice guideline, the investigator needed to measure all recommended behaviors outlined in the guideline, identify the percentage of recommended behaviors that signified research use, or illustrate how outcomes reflected their conceptualization of research use. If this was not done, we could not be certain the investigators were measuring research use and so we excluded the study.

### Screening Process

**Figure 1:**
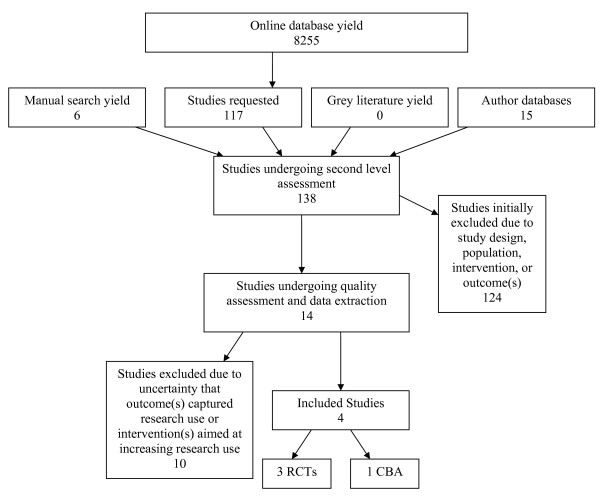
Search and retrieval process.

The search resulted in over 8,000 titles. One author reviewed titles, abstracts and selected studies. Two reviewers each screened 20% of the titles and abstracts. Inter-rater reliability between reviewers was greater than 90%. The initial screening process resulted in 117 studies. Manual and ancestry searching produced an additional 21 studies. Further review of the 138 studies narrowed them to 14 and the final result was four studies meeting the inclusion criteria [24-27]: three RCTs and one CBA (Figure 1).

### Excluded Studies

In the final exclusion of studies, ten of the studies were excluded for two reasons: uncertainty that the outcomes were measuring research use [28-31], and interventions not explicitly aimed at increasing research use or evidence-based practice [32-37] (Table 2).

**Table 2:** Details of excluded studies

First Author	Description of Study Purpose	Reason for Exclusion from Review
Davies ^[28]^	To determine whether using a specific intervention would lead to more appropriate implementation of guidelines	1. Investigators do not describe guideline content and recommendations
		2. Investigators do not specify what percentage or number of guideline recommendations must be met to signify effectiveness
		3. Unable to determine the extent guideline recommendations were followed
Hodnett ^[29]^	To evaluate the effectiveness of an intervention to promote research-based nursing care	1. Investigators described the content of the intervention but the outcomes do not correspond to the content
		2. Unable to determine how the outcomes represent research use
McDonald ^[30]^	To test the effectiveness an intervention to increase nurses adherence to pain assessment and management guidelines, and to improve patient outcomes	1. Investigators do not specify what percentage or number of recommendations must be met to signify effectiveness
		2. Investigators do not measure all recommendations of the intervention
		3. Unable to determine the extent of recommendation adherence
Murtaugh ^[31]^	To test the effectiveness of two interventions designed to improve the adoption of evidence-based practices	1. Investigators do not specify what percentage or number of recommendations must be met to signify effectiveness
		2. Investigators do not measure all recommendations of the intervention
		3. Unable to determine the extent of recommendation adherence
Feldman ^[32]^	To assess the impact and cost-effectiveness of two interventions designed to improve management and outcomes of patients	1. Not explicitly aimed at increasing research use or evidence-based practice
Feldman ^[33]^	To examine the effect of an intervention designed to standardize nursing care, strengthen nurses' support for patient self management, and yield better patient outcomes	1. Not explicitly aimed at increasing research use or evidence-based practice
Gould ^[34]^	To develop, implement, and evaluate an intervention designed to promote nurses' compliance with key procedures	1. Not explicitly aimed at increasing research use or evidence-based practice
		2. Unable to determine if 'key procedures' are evidence-based
Jones ^[35]^	To develop and test an intervention to improve practices, knowledge, attitudes, and policies	1. Not explicitly aimed at increasing research use or evidence-based practice
Moongtui ^[36]^	To evaluate the effectiveness of an intervention on nursing practices	1. Not explicitly aimed at increasing research use or evidence-based practice
Krichbaum ^[37]^	To test the effectiveness of interventions designed to improve patient outcomes	1. Not explicitly aimed at increasing research use or evidence-based practice

### Methodological Quality

We evaluated the studies for methodological quality using two tools available from the Cochrane Collaboration Effective Practice and Organization of Care Group (EPOC) [38]. The RCT tool consisted of items related to unit of analysis, power, baseline measure, concealment of allocation, blinded or objective assessment of outcome(s), protection against contamination, reliable outcome(s), and completeness of follow-up. The CBA tool consisted of items related to unit of analysis, power, baseline measure, comparability of groups, blinded or objective assessment of outcome(s), protection against contamination, reliable outcome(s), and completeness of follow-up. In both tools, unit of analysis errors were determined using the unit of allocation and unit of analysis items. That is, if authors allocated by cluster and analyzed by individual without reporting appropriate statistical measures to account for clustering, we reported unit of analysis errors. If in these cases the authors reported power calculations and did not account for intra-cluster correlations, we scored the power calculation item as done but accounted for the error in the overall rating. We report results in Table 3.

**Table 3:** Methodological quality of included studies

CBA Methodological Quality Assessment Results and Rating											
First Author	Unit of Allocation	Unit of Analysis	Power Calculation	Baseline Measure	Characteristics of Control	Blinded Outcome Assessment	Protection Against Contamination	Reliable Outcomes Measure	Provider Follow	Patient Follow Up	Rating

Tsa i ^[27]^	Provider	Provide r	NC	√	√	X	NC	X	√	n/a	Low

RCT Methodological Quality Assessment Results and Rating											

First Author	Unit of Allocation	Unit of Analysis	Power Calculation	Baseline Measure	Allocation Concealment	Blinded Outcome Assessment	Protection Against Contamination	Reliable Outcomes Measure	Provider Follow Up	Patient Follow Up	Rating

Dufault ^[24]^	Ward	Provider *	NC	X	NC	X	√	X	NC	n/a	Low
Hong ^[25]^	Ward	Provider *	NC	√	√	X	√	X & NC	NC	n/a	Low
Tranme r ^[26]^	Ward	Provider *	NC	X	NC	X	√	X	NC	n/a	Low

√:Done

X: Not Done

NC: Not Clear

* Unit of analysis error

4/8 or less – low quality

5/8–6/8 – medium quality

7/8 or higher – high quality

Two reviewers assessed each study and discrepancies were resolved through discussion. Each item was scored as: done, not done, and not clear. A quality rating was assigned to each study as low, medium, or high depending whether it scored done on zero to four, five to six, or seven to eight items respectively. Unit of analysis errors and incorrect power calculations were noted. We did not use quality assessment ratings to exclude studies because we sought to explore the general state of the science in this field.

### Data Extraction

We extracted data from four studies representing five experimental cohorts where an intervention was compared to a control. One reviewer independently extracted data from all four studies while two reviewers each extracted data from two of the studies. We used extraction tools and dictionaries available from EPOC [38]. Data on design, subjects, setting, interventions and outcomes were extracted.

To facilitate comparison and discussion, we classified interventions using an EPOC classification system [38]. Interventions were classified as: educational meetings, multidisciplinary committees and local opinion leaders. The EPOC classification is used throughout the text (Table 4).

**Table 4:** Outcome measure and classification of research utilization intervention

Author/Year/Country	Study Design	Setting and Specialty	Description of Intervention(s)	Classification Using EPOC Method	Outcome Measure
Dufault, 1995 United States ^[24]^	RCT	Hospital/Oncology	1. Organization of practitioners and researchers aimed at solving a clinical problem using research findings	1. Multi-disciplinary team	Kim's Research Utilization Competency Scale ^[39]^
Hong 1990 China ^[25]^	RCT	Hospital/Inpatient	1. In-service education and demonstration tutorial by opinion leader	1. Educational meetings	Compliance with all clinical practice guideline recommendation s
				2. Local opinion leaders	
Tranme r2002 Canada ^[26]^	RCT	Hospital/Medical & Surgical	1. Workshops about conducting a research study and using the findings	1. Educational meetings	Champion and Leach Research Utilization Questionnaire ^[40-41]^
			1. Workshops about research findings	1. Educational meetings	Champion and Leach Research Utilization Questionnaire ^[40-41]^
Tsai, 2003 Taiwan ^[27]^	CBA	Hospital/Inpatients	1. Workshops about research utilization	1. Educational meetings	Tsai Research Utilization Questionnaire

Several studies in this review reported additional outcomes, for example, on predictors of research use, changes in knowledge or attitudes, or patient outcomes. These were not extracted or reported on as we did not consider them as measures of research use *per se*.

## Results

### Methodological Quality of Included Studies

Overall, the quality of the studies was low (Table 3). Two had unit of analysis errors where the investigators allocated by group but did not account for clustering in the analysis [24,25]. Of the two studies without unit of analysis errors, the investigators of one study allocated by unit and accounted for clustering [26], while the other allocated and analyzed at the provider level [27]. No authors presented power calculations. Two studies had substantial differences in outcomes prior to the intervention [24,26]. Allocation concealment was not reported in two RCTs [24,26]. None of the investigators used blinded or reliable outcome assessments. The CBA investigators did not protect against contamination of the intervention across study groups [27]. However, the RCT investigators all randomized by ward and attempted to protect against contamination [24-26]. The CBA investigator reported adequate provider follow up [27]. However, the RCT investigators either used separate samples [25,26], or did not report on follow up [24].

### Included Studies

Four studies representing five intervention cohorts in Canada, USA, Taiwan, and Hong Kong met our inclusion criteria (Table 4). Three were RCTs (four intervention cohorts) [24-26], and one was a CBA (one intervention cohort) [27]. All studies included nurses from inpatient clinical settings; oncology, medicine, surgery and multiple specialties.

Investigators assessed educational meetings delivered to nurses in three studies [25-27]. In one study, the investigators compared two investigator-provided educational interventions to a control [26]. Because these interventions varied in content and duration, we identified this study as having two cohorts. Another study used a combination of local experts and educators to deliver the intervention [27]. The third study that assessed educational meetings used local opinion leaders identified by the study participants to conduct a demonstration tutorial which was supplemented with education delivered by a local expert [25]. One study investigated the formation of a multidisciplinary team of practitioners and researchers [24]. Within this intervention there were components of education and marketing. However, the investigators based their conclusions on the entire intervention (the multidisciplinary team) rather than the components, therefore, we did not separate the components of this intervention.

The investigators of three studies used nurse-administered instruments to measure research use. Dufault [24] used Kim's [39] 13-item Likert-type scale that asked participants to rate their research utilization competency on a one to seven scale. Tranmer [26] used the Research Utilization Questionnaire (RUQ) developed by Champion and Leach [40,41]. This 42-item Likert-type questionnaire measured attitudes towards research, access to research, support of the use of research and research use. The questionnaire was divided into corresponding subscales. Because Tranmer [26] reported and analyzed the results of each subscale, we extracted only the data that pertained to the use of research subscale. Finally, using an instrument based on her previous work, Tsai [27] assessed whether research utilization was implemented in nursing practice and to what degree. Tsai's instrument consisted of 11 items including single-choice, multiple-choice and open-ended questions.

In the final study by Hong, investigators used self-reporting and participant observation to assess practice compliance with all the recommendations from a clinical practice guideline [25]. This study differed from many of the excluded studies that assessed provider behavior change. Specifically, the investigators linked all eight outcomes to the eight practices recommended by the clinical guideline, which was referenced to research, thus providing support that the outcomes did reflect research use.

### Findings

Methodological weaknesses, varied interventions and outcomes across health contexts, incomplete reporting, and the small samples prevented meta-analysis. Instead, we present narrative results. The characteristics and findings of the four studies included in this review are summarized in Tables 5 and 6. All findings must be interpreted with significant caution given the low quality of studies.

**Table 5:** Effect of interventions on research use

First Author	Intervention(s)	Outcome(s) of Interest	Effect of Intervention(s) on Outcome(s) of Interest
Dufault ^[24]^	Multidisciplinary team	1. Kim's research utilization competency scale ^[39]^	Significant change
Hong ^[25]^	Educational meetings led by local opinion leader	1. Proportion of reported catheter practices meeting guidelines recommendations	Significant change
		1. Proportion of observed catheter practices meeting guideline recommendations	Significant change
Tranmer ^[26]^,	Educational meetings #1	1. Champion and Leach Research ^[40-41]^Use Questionnaire	No significant change
Tranmer ^[26]^	Educational meetings #2	1. Champion and Leach Research ^[40-41]^Use Questionnaire	No significant change
Tsai ^[26]^	Educational meetings	1. Tsai Research Utilization Questionnaire.	No significant change

**Table 6:** Characteristics of included studies and detailed description of intervention

First Author	Study Subjects	Deliverer/Recipient of Intervention	Length of Intervention (Dose)	Detailed Description of Intervention
Dufault ^[24]^	27 nurses from 4 oncology units	Both nurses and researcher s/nurses	28 weeks consisting of 6 sequential phases	Nurses and investigators participated in activities related to optimal pain management. The phases included:
				1. Problem identification and assessment of research bases for utilization
				2. Evaluation of research relevancy to problem selection, nursing department values, standards and policies, and potential cost and benefit
				3. Innovation design to meet the needs of the problem within the scope of the research base.
				4. Actual or construct replication and evaluation of the innovation.
				5. Decision to adopt, alter or reject the innovation.
				6. Development of means to extend the innovation within and outside of the setting.
Hong ^[25]^	220 nurses surveyed/255 episodes of care observed from 3 medical and 3 surgical units	Local opinion leaders and infection control nurses/Nurses and student nurses	30 minute lecture and unspecified length demonstratio n tutorial	Infection control nurses provided lectures on research based practices surrounding catheter care. Local opinion leaders provided demonstration tutorials to group of 6–10 nurses following the lectures.
Tranme r ^[26]^	235 nurses from 6 medical/surgical units	Researchers/nurses	20 hours for 'high' intervention and 8 hours for 'low' intervention	High intervention: Nurses learned how to review and critique research literature, completed a literature review on a clinical practice, participated in the design of a research study to address the identified clinical problem, and participated in the implementation of the study.
				Low intervention: Nurses learned about the literature related to a clinical problem and discussed now best to implement the research study.
Tsai ^[27]^	89 nurses from multiple clinical units	Clinical experts/nurses	65 hour workshops delivered over 8 weeks	Research utilization education designed and based on steps of research utilization:
				1. Preparation stage
				2. Confirmation stage
				3. Comparison and
				assessment stage
				4. Decision stage
				5. Implementation stage
				6. Evaluation stage

### Educational meetings

Two studies representing three cohorts tested the effect of interactive educational meetings on research utilization [26,27]. Tranmer measured research use both in nurses who participated and nurses from the same unit as those who participated [26]. There were no significant changes in research utilization scores in either group. This suggests that, based on this study, educational meetings are ineffective whether a nurse participates directly (attending education meetings) or indirectly (working with nurses who attended educational meetings but not attending themselves). However, no definite conclusions can be drawn due to design limitations.

Educational meetings of varying content, frequency and duration (Table 6) were also found to be ineffective. Tranmer, who did not describe frequency of their intervention, reported non-significant changes in research utilization scores regardless of whether the intervention was twenty hours and focused on literature critiquing, research design, and protocol implementation, or eight hours and focused solely research design and implementation [26]. These results are supported by Tsai's study, in which she tested a series of educational strategies focused on research use totaling 65 hours and delivered over eight weeks [27].

Interactive educational meetings did not have a delayed effect on research utilization. Tsai measured research use at two points: immediately and six months following the intervention. In both cases, there were no significant changes in research utilization [27]. Similar findings were reported by Tranmer who measured research utilization only once, one year following the start of the intervention and also reported non-significant results [26].

In summary, based on this review, educational meetings of varying content, duration, and frequency cannot be said to be effective research utilization interventions in nursing. The studies were few in number and were of poor quality. Clearly, there is inconclusive evidence and educational meetings require more rigorous investigations to determine their effect in nursing.

### Educational meetings and local opinion leaders

One study tested the effect of interactive educational meetings combined with a local opinion leader, and found that nurses who attended both the lecture and the tutorial (led by a local opinion leader) reported increased research utilization related to urinary catheter practices [25]. It was not possible to determine whether the positive effect was due to the local opinion leader, the educational meeting, or a combination of both. The intervention consisted of a 30 minute lecture by an educator, followed one week later by a demonstration tutorial conducted by a local opinion leader (Table 6). The length of the demonstration tutorial was not reported. No data were collected during the lapse between interventions. Outcomes were assessed twice: two weeks and two months following the intervention. The authors used a practice survey at two weeks, and direct observation at two months. Longitudinally, education and local opinion leaders appeared to sustain an increase in research utilization, but this study was also of low quality and represents inconclusive evidence for educational interventions combined with a local opinion leader.

### Multidisciplinary committees

One study was found in which formation of multidisciplinary committees was reported to be effective at increasing nurses' research use related to oncology pain [24]. The intervention lasted 28 weeks and was divided into six stages (Table 6). Each stage was sequential and lasted between two and nine weeks. Stages were constructed around collaboration of members of the multidisciplinary team working to operationalize an existing research utilization process (the Conduct and Utilization of Research in Nursing Project) [42]. Unlike other interventions, education was not the primary component. Outcomes were assessed at one point using a research utilization scale. The investigators did not report the duration between the intervention and outcome measurement. Multidisciplinary committees require further investigation.

### Summary of Findings

In summary, the four studies reviewed were of poor quality [24-27]. The findings of this review represent a lack of evidence to support or refute the benefit of educational meetings for increasing research utilization in nursing, and further study is required.

## Discussion

Study design and implementation must improve before one can comment on the effectiveness of interventions aimed at increasing research use in nursing practice. The current state of the science provides little guidance to individuals charged with implementing strategies to increase research use in nursing practice. We now relate our findings to current literature and provide a discussion of conceptual and methodological challenges facing the field.

### Comparison with Existing Reviews

In a review of organizational interventions aimed at increasing evidence-based nursing practice, Foxcroft and Cole could locate no rigorous studies [11]. Grimshaw and colleagues [6-8] published comprehensive reviews of provider behavior change reviews and guideline dissemination strategies. While we were interested specifically in nurses' research utilization and Grimshaw and colleagues [6-8] examined broader outcomes (provider behavior change and guideline dissemination), these reviews were all aimed at improving understanding of how to translate research findings into practice. Grimshaw and colleagues [7] concluded that interventions with different educational strategies showed mixed effects depending upon a combination of strategies. We report inconclusive evidence compared to these results [24-27]. The educational interventions included in our review were small interactive group sessions. In medicine, these types of educational strategies showed the most promise. We did find some limited support for two interventions: multidisciplinary committees and local opinion leaders. Grimshaw and colleagues [7] also found that multidisciplinary collaboration was effective and that use of local opinion leaders showed mixed effects.

Similarities and differences between these reviews can be attributed to multiple factors. Perhaps the most obvious is in the review methods. Grimshaw and colleagues [8] had a more robust dataset and derived a single effect size for each of the 235 studies reviewed, as well as summarizing the range of effects and median effects across studies for each intervention. In contrast, we were only able to locate four studies, and were limited to a narrative analysis based on the number of positive and negative results (vote counting).

Secondly, investigators of existing reviews have focused on changing provider behavior [6,7], implementing practice guidelines [8,10] and conducting continuing education [9]. We located no review focusing specifically on research utilization. Instead, authors have relied upon changes in patient outcomes, provider behavior, or a combination of both. For example, in a systematic review of guidelines in professions allied to medicine, 24% of nursing studies measured patient outcomes, 24% measured provider behavior, and 53% measured both provider and patient outcomes [10]. Additionally, all four nursing studies included in a review of continuing education and workshops measured provider outcomes [9]. Findings from these reviews [6-10] are increasingly being used to guide strategies aimed at increasing research use in clinical practice [13]. Hakkennes and Green suggested that authors must choose between assessing changes in provider practice or changes in patient outcomes when evaluating interventions aimed at implementing evidence or changing practice [13]. While this is true for the latter, we argue there is a third and perhaps more accurate choice: assessing changes in research use. By not assessing research use and moving directly to changes in provider or patient outcomes, investigators are treating research utilization interventions as a 'black box' phenomenon that somehow produces a change in clinician behavior or patient outcomes. Clearly, there are additional factors that influence both clinician and patient outcomes. The assumption that research use is the only mediating factor represents a large gap in the literature, and thus our understanding of how to increase the use of research in practice.

### Conceptual Challenges

A major conceptual issue we identified is related to outcome measurement. We excluded several studies due to unclear conceptualizations of research use (Table 2). Investigators have commonly aligned themselves with a model of evidence-based practice consisting of five steps: 1) converting information needs to an answerable question, 2) locating the evidence, 3) critically appraising the evidence, 4) implementing the evidence in practice, and 5) evaluating care performances [43]. Most often, evaluations of interventions designed to increase evidence-based practice (or in our case, research use) relied upon behavior change outcomes [13]. Using the model outlined above, this approach is synonymous with evaluating care performances (Step five). However, research use is only one of the factors that influence care performance or behavior change [21-23]. Reliance on behavior change outcomes has treated the implementation stage (Step four) also as a 'black box' and led to less understanding of how implementation of research in practice occurs. We and others argue that the key to increasing research use in practice lies in the implementation stage [6,22,44,45].

There is a lack of clarity and uncertainty, in fact a near silence, in the research community about what constitutes an appropriate measure of research use [46-48]. This silence can be partly to blame for a lack of research examining the intervention stage of research use and can be attributed to a poor understanding of the conceptual structure of research utilization [46,49]. Ideally, selecting outcomes to assess the effectiveness of an intervention aimed at increasing research use should be informed by an explicit conceptualization of research use [22,50]. Only two authors in our review explicated how they conceptualized research utilization [24,26]; both offered different conceptualizations, and it was not clear from either how their conceptualization informed outcome selection. Rich argues that misconceptions of how research-based knowledge enters the decision-making process lead to inaccurate measures of research use [51]. Estabrooks and colleagues suggested that "unresolved measurement challenges present an important and practical problem" to advancing the field of research utilization [22]. The findings of this review support these claims and suggest such issues persist.

Investigators are interested in the link between using research in practice and improving patient outcomes. The abundance of studies focusing on behavior change and patient outcomes as a result of research uptake points at this interest. However, establishing this link is best accomplished if we first develop sufficient evidence to support the relationship between specific interventions and research use. From this, we can explore the relationship between effective research use interventions and behavior change or patient outcomes. If studies aim to evaluate an intervention to increase research use, outcomes must be structured to capture changes in research use. More attention to the fit between study outcomes and the conceptual structure of research use will advance the field by peering into the 'black box' and producing more accurate results.

Common problems with the instruments used in the studies we reviewed, and elsewhere, include lack of theory (measurement or research utilization theory), lack of construct clarity, lack of psychometric assessment, a presumption of linearity, lack of longitudinal work, and influential yet unacknowledged assumptions [22]. Until more reliable and valid instruments are developed, investigators should present explicit statements outlining the conceptual and practical basis for chosen outcomes. Making use of available conceptualizations to operationalize research use would increase the validity and ability to compare results across studies [16,17], [51-53].

### Methodological Challenges

The studies in our review were published between 1990 to 2003. Methodological quality (Table 3) was low in all four [24-27]. This suggests that the field is not developing within nursing as would be expected. We present what we believe are the most urgent methodological challenges facing the field.

### Identification of Primary Outcomes

A primary outcome helps determine the key endpoint signifying the effectiveness of an intervention [54]. Explicit reporting of the primary outcome enables the reader to determine whether the study results provide sufficient evidence for an intervention and to whom the study results apply. In our review, we extracted only research use outcomes. However, three investigators in this review also reported outcomes additional to research use and all three assessed attitude towards research [24,26,27]. The relationship between such characteristics and research use is not well-supported [55]. When authors report on multiple outcomes without discussing why particular measures were chosen or what constitutes the primary outcome, it is difficult to interpret study findings in the context of research utilization.

### Use of Multiple Outcomes

The challenge in using multiple outcomes to evaluate research utilization interventions is determining the number that must be changed to indicate effectiveness [33]. We excluded many studies due to uncertainty that the investigators were actually measuring research use (Table 2). In these cases, rationale or support for multiple outcomes in the context of research utilization was not provided. It is challenging to determine whether an intervention was effective at increasing research use if there were changes in some, but not all, of the outcomes. Also challenging is determining how many recommendations from clinical practice guidelines must be met to indicate research use, and for this review we included only one study that measured all recommended practices [25]. Measuring all outcomes may not be the most accurate or feasible approach, especially if guidelines recommend large numbers of practices or procedures.

### Intervention Sustainability

Two studies [24,27] measured longitudinal outcomes; one illustrated a benefit of intervention over time (two months) [24] and the other illustrated no effect either immediately or six months following [27]. Longitudinal outcome measurements will advance our understanding of the optimal timing and frequency of outcome evaluation and are needed to establish the sustainability of research use. Titler has described two challenges in assessing sustainability of research utilization interventions: defining the boundary between the end of the intervention phase and the start of the sustainability phase, and timing the outcome measurement to differentiate between sustained improvements and residual effects [48]. Compartmentalizing these stages becomes difficult when multiple and overlapping interventions are tested. Thus far, the literature on research utilization provides little guidance on the optimal timing or length of outcome measurement for different interventions. Hong [25] and Tsai [27] did not report why they assessed outcomes at two and six months. Future investigators should clearly describe intervention characteristics such as duration and frequency, deliverer and receiver, and mode of delivery. Guidelines such as the Consolidation of Standards for Reporting of Trials (CONSORT) [56] or the CONSORT statement for cluster RCTS [57] should be followed. Future reviews would also benefit from using a common classification system for interventions. We used a classification system proposed by EPOC. However, this approach may require adaptation for use in nursing and needs to be examined and validated.

### Unit of Analysis Errors

Two RCTS included in our review had unit of analysis errors (Table 3) [24,25]. Unit of analysis errors occur when investigators assign clusters or groups of individuals to a study group (i.e., intervention or control), and then analyze as if each individual had an equal chance of being assigned to either group [58]. When this occurs, outcomes for each individual are not independent of others within the same group. This is a unit of analysis error because people within clusters share similarities (i.e., burn unit and psychiatric nurses are each familiar with different practices) that may not be accounted for during analysis. When clustering is ignored, the number of participants required (sample size) is underestimated and the level of study significance (p value) is overestimated, resulting in an over-estimate of the precision of the result [59]. Future studies should be designed and analyzed using methods that account for clustering if allocation is done by groups of individuals, and allocation procedures should be unbiased (i.e., central randomization) and explicitly outlined in study reports.

### Limitations

This systematic review has some limitations. First, we did not conduct a meta-analysis because of lack of effect sizes and a small size. The method we used (vote counting) is a crude estimate of effectiveness. Second, we used the EPOC classification that was developed for broad use [38]. Its applicability specifically to nursing has yet to be established. Third, the four studies included were all of low quality. Including studies of low quality limits any conclusions. Fourth, we included only studies published in English. While researchers have reported that language restrictions do not change the results of systematic reviews on conventional medicine interventions [60,61], the impact of language restriction on research utilization intervention systematic reviews is unknown. However, it is possible that we missed potential studies due to our language restriction. Finally, because we focused on research use as the outcome of interest specific to intervention effectiveness, comparisons between our review and those of others must be made cautiously.

## Conclusion

Little is known about how to increase research use in nursing and we currently lack evidence to either support or refute the effectiveness of specific interventions. Advancing the field of research utilization interventions in nursing requires methodological and conceptual advancement. If investigators aim to establish a link between using research and improved patient outcomes they must first establish those interventions that are effective at increasing research use.

## Competing interests

The author(s) declare that they have no competing interests.

## Authors' contributions

CE, SSF and LW conceived the study. CE, SSF, LW and KM supervised DT's thesis. SSF and LW validated the search design, article selection, data extraction, and quality assessment. CE, SSF, and LW validated the analysis. DT designed and performed the search, selected the articles, assessed the quality of included studies, extracted the data, analyzed the results and wrote the manuscript. CE, SSF, LW, and KM reviewed the paper and participated actively throughout the writing of the paper. All authors read and approved the final manuscript.
